# B-mode and colour Doppler sonographic examination of the milk vein and musculophrenic vein in dry cows and cows with a milk yield of 10 and 20 kg

**DOI:** 10.1186/1751-0147-54-15

**Published:** 2012-03-13

**Authors:** Ueli Braun, Eva Forster

**Affiliations:** 1 Department of Farm Animals, Vetsuisse Faculty, University of Zurich, Zurich, Switzerland

## Abstract

**Background:**

This study investigated the effect of milk yield on blood flow variables in the milk vein and musculophrenic vein in dairy cows.

**Methods:**

Five healthy dry cows, five cows with a daily milk yield of 10 kg and five others with a daily milk yield of 20 kg underwent B-mode and colour Doppler sonographic examination. The diameter of the veins, blood flow velocities and blood flow volumes were measured on both sides in standing, non-sedated cows using a 7.5 MHz linear transducer.

**Results:**

Lactating cows had significantly higher blood flow velocities in the milk vein than dry cows; the maximum blood flow velocity of dry cows and those with a daily milk yield of 10 and 20 kg were 14.04, 38.77 and 39.49 cm/s, respectively, the minimum velocities were 0.63, 3.02 and 2.64 cm/s, respectively, and the mean maximum velocities were 8.21, 26.67 und 28.22 cm/s, respectively. Cows producing 20 kg of milk a day had a blood flow volume of 3.09 l/min, which was significantly higher than 0.79 l/min recorded in dry cows. Lactating cows had significantly higher mean maximum blood flow velocities in the musculophrenic vein than dry cows. Blood flow variables of both veins did not differ significantly between the left and right side.

**Conclusion:**

This study showed that milk yield has a profound effect on blood flow variables in the milk vein and to a lesser extent the musculophrenic vein. This must be taken into consideration in future Doppler sonographic studies of these veins and possibly other vessels. Furthermore, measurements on one side are representative of both sides.

## Background

The ultrasonographic characteristics of various arteries and veins in cattle have recently been described using colour Doppler [1-3]. The milk vein and musculophrenic vein are particularly amenable to Doppler ultrasonographic examination because of their superficial location, and cows tolerate examination of these veins better than the jugular vein [4]. The milk vein drains blood from the udder. However, it is not known whether Doppler sonographic characteristics of blood flow vary with the amount of milk produced or whether the stage of lactation needs to be considered when interpreting results. The present study tested the hypothesis that milk yield has an effect on Doppler sonographic findings of the milk vein but not of the musculophrenic vein because the latter drains blood from the costal parts of the diaphragm and from the wall of the thorax and abdomen [5]. To achieve this, five dry cows, five cows that produced 10 kg of milk per day and five others that produced 20 kg of milk per day underwent Doppler sonographic examination of the milk vein and musculophrenic vein. A secondary goal was to determine whether blood flow variables in these two veins differed between the left and right sides, as has been reported in goats [6]. The findings of this study would be useful for the interpretation of previously established normal ranges of blood flow variables of the milk vein and musculophrenic vein [1-3] in relation to milk yield.

## Methods

The study protocol was approved by the Animal Care Committe of the Canton of Zurich, Switzerland.

### Animals

Fifteen healthy Swiss Braunvieh cows between 2.5 and 17 years of age (median, 8 years) were used. Group 1 included five cows that had been dry for at least 4 weeks and were at least two weeks from the due date. Group 2 included five cows with a daily milk yield of 10 kg (9.4 to 12.0 kg; mean ± sd, 11.0 ± 1.4 kg) and group 3 included five cows with a daily milk yield of 20 kg (18.4 to 22.0 kg; 20.5 ± 1.7 kg).

### Clinical examination

Clinical examination included assessment of demeanour and general condition, appetite, defaecation and urination, rectal temperature, heart and respiratory rates, ruminal motility and intestinal sounds. The cows were weighed and examined on both sides by simultaneous percussion and auscultation. A urine sample was examined for glucose, protein, ph, ketones, nitrite, leukocytes, bilirubin, blood, urobilin using a test strip (Combur9-Test^®^, Roche, Basel). The udder was examined visually and palpated, and a California mastitis test was carried out in the lactating cows. All cows were clinically healthy and weighed between 500 and 795 kg (677.9 ± 84.8 kg, mean ± sd). There was no significant differences among the three groups.

### B-mode and colour Doppler ultrasonography

The examinations took place between 8:00 and 10:00 in the morning and 0.5 to 2.5 h after milking in lactating cows. B-mode and colour Doppler ultrasonographic examination of the milk vein (subcutaneous abdominal vein) and musculophrenic vein were carried out in all cows on the left and right sides. The procedure for B-mode and colour-Doppler ultrasonography has been previously described in detail [2,3,7]. The hair over the caudal part of the left and right milk veins was clipped to yield a 20-cm length for assessment, which took place in area immediately cranial to the udder or up to 15 cm cranial to the udder. For the examination of the musculophrenic vein, a 50 cm × 50 cm area in the sternal region was clipped. The examination took place near the sternum from the 5th and 6th intercostal spaces and slightly ventral to the point of the elbow.

A 7.5 MHz real-time linear transducer (Hitachi Ultrasound scanner EUB 8500) was used to examine the veins. A stand-off with an additional angular correction was used to maintain an angle of no more than 60° between the transducer and the vein. During ultrasonographic examination, pressure exerted by the transducer on the milk vein was kept to the absolute minimum that was required for sufficient contact between transducer and skin. The veins were first examined in longitudinal and cross section using B-mode. After freezing the ultrasonographic image, the diameter of the veins was measured electronically in cross section from intima to intima of the vessel using the electronic cursors. Then the distance between the vein and the skin surface was measured. The colour-Doppler gate was directed parallel to the wall of the vein to visualise the blood flow and then positioned at a 60° angle in the centre of the milk vein. By turning on the pulsed-wave colour-Doppler ultrasound instrument, a sound could be heard and the spectral display was visible on the screen. Optimal colour flow and spectral curve images were frozen on the screen to determine the maximum, minimum and mean blood flow velocities and the blood flow volume during a measurement period of 2 min.

### Statistical analysis

The program StatView 5.1 (SAS Institute Inc., Cary, NC) was used for statistical calculations. Frequencies, means and standard deviations were calculated and differences were analysed using analysis of variance (ANOVA) and an unpaired two-sided *t*-test for normally distributed variables. Pairwise analysis of factorial ANOVA was performed according to the post hoc test by Bonferroni-Dunn. A value of P < 0.05 was considered significant.

## Results

### B-mode and colour Doppler sonography of the milk vein

In B-mode, the milk vein appeared as a vessel immediately beneath the skin as described previously [2]. The spectral display in Doppler mode was a broad band structure with varying band width and a wave-like course also as described previously [2]. The distance between the vein and the skin surface and the diameter of the vein did not differ among the three groups and ranged from 0.44 to 0.53 cm and from 1.88 to 1.99 cm, respectively (Table 1). The blood flow velocities in groups 2 (10 kg) and 3 (20 kg) were significantly greater than in group 1 (dry cows) (*P *< 0.05). Maximum velocities in groups 1, 2 and 3 were 14.04, 38.77 and 39.49 cm/s, respectively, the minimum velocities were 0.63, 3.02 and 2.64 cm/s, and the mean maximum velocities (mean of the maxima recorded during individual measuring periods of 2 min) were 8.21, 26.67 and 28.22 cm/s. In group 3 the blood flow volume was significantly larger than in group 1 (3.09 l/min versus 0.79 l/min, *P *< 0.05).

**Table 1:** Blood flow variables in the milk vein of 5 dry cows, 5 cows with a daily milk yield of 10 kg and 5 cows with a daily milk yield of 20 kg (mean, sd, min., max.)

	Group		
	
Variable	Dry	10 kg milk	20 kg milk
Distance between vein and skin surface (cm)	0.53 ± 0.12 (0.37 - 0.64)	0.45 ± 0.11 (0.33 - 0.63)	0.44 ± 0.05 (0.39 - 0.50)

Diameter of vein (cm)	1.93 ± 0.39 (1.47 - 2.54)	1.88 ± 0.77 (1.30 - 2.97)	1.99 ± 0.46 (1.52 - 2.58)

Maximum blood flow velocity (cm/s)	14.04 ± 2.04 (12.0 - 16.8)	38.77 ± 15.17* (15.6 - 56.1)	39.49 ± 8.28*(28.5 - 50.6)

Minimum blood flow velocity (cm/s)	0.63 ± 0.26 (0.3 - 0.9)	3.02 ± 1.53* (1.0 - 5.1)	2.64 ± 0.61* (2.0 - 3.6)

Mean maximum blood flow velocity (cm/s)	8.21 ± 1.69 (6.5 - 10.6)	26.67 ± 11.91* (8.8 - 40.6)	28.22 ± 5.41*(21.7 - 33.0)

Blood flow volume (l/min)	0.79 ± 0.29 (0.4 - 1.2)	1.80 ± 0.72 (1.3 - 3.0)	3.09 ± 1.46* (1.8 - 5.3)

* Different from dry cows, *P *< 0.05

### B-mode and colour Doppler ultrasonography of the musculophrenic vein

The musculophrenic vein could be visualised in all the cows and appeared as a vessel running parallel to the longitudinal axis of the animal within the diaphragmatic musculature as described previously [3]. The spectral display appeared also as described [3] as a broad band with a wave-like course. The distance between the vein and the skin surface and the diameter of the vein did not differ among the three groups and varied from 0.97 cm to 1.18 cm and from 0.63 to 0.87 cm, respectively (Table 2). The blood flow velocities of groups 2 and 3 were greater than the velocity of group 1, but only the differences between the mean maximum velocities were significant. The values of the mean maximum velocity for groups 1, 2 and 3 were 30.59 cm/s, 81.90 cm/s and 85.15 cm/s, respectively. The blood flow volume increased numerically from group 1 (0.52 l/min) to group 2 (0.70 l/min) to group 3 (1.57 l/min) but the differences were not significant.

**Table 2:** Blood flow variables in the musculophrenic vein of 5 dry cows, 5 cows with a daily milk yield of 10 kg and 5 cows with a daily milk yield of 20 kg (mean, sd, min., max.)

	Group		
	
Variable	Dry	10 kg milk	20 kg milk
Diameter of vein (cm)	0.69 ± 0.13 (0.28 - 1.10)	0.63 ± 0.09 (0.38 - 0.87)	0.87 ± 0.15 (0.54 - 1.32)

Maximum blood flow velocity (cm/s)	61.78 ± 12.8 (30.0 - 94.9)	118.08 ± 19.9 (77.6 - 189.5)	119.13 ± 16.0 (78.8 - 159.8)

Minimum blood flow velocity (cm/s)	1.96 ± 0.40 (0.8 - 3.1)	8.80 ± 2.80 (2.9 - 18.4)	7.87 ± 1.40 (3.4 - 11.0)

Mean maximum blood flow velocity (cm/s)	30.59 ± 5.6 (15.1 - 43.5)	81.90 ± 16.3* (45.2 - 136.3)	85.15 ± 12.3*(59.6 - 116.5)

Blood flow volume (l/min)	0.52 ± 0.20 (0.0 - 1.5)	0.70 ± 0.10 (0.5 - 0.9)	1.57 ± 0.40 (0.8 - 3.1)

* Different from dry cows, *P *< 0.05

### Comparison of blood flow variables from the left and right side of the body

The blood flow variables calculated for the milk vein and musculophrenic vein did not differ significantly between the two sides of the body (Table 3).

**Table 3:** Comparison between blood flow variables of the milk vein and musculophrenic vein recorded on the left and right side of 5 cows (mean, sd, min., max.)

Vessel	Variable	Left	Right
Milk vein	Distance between vein and skin surface (cm)	0.48 ± 0.10 (0.33 - 0.64)	0.44 ± 0.09 (0.32 - 0.56)
	
	Diameter of vein (cm)	1.93 ± 0.52 (1.30 - 2.97)	1.86 ± 0.47 (1.41 - 3.00)
	
	Maximum blood flow velocity (cm/s)	30.77 ± 15.4 (11.9 - 56.1)	37.21 ± 15.3 (11.0 - 58.9)
	
	Minimum blood flow velocity (cm/s)	2.10 ± 1.4 (0.3 - 5.0)	2.15 ± 1.0 (0.6 - 3.7)
	
	Mean maximum blood flow velocity (cm/s)	21.03 ± 11.8 (6.5 - 40.5)	24.59 ± 11.9 (6.6 - 37.9)
	
	Blood flow volume (l/min)	1.90 ± 1.30 (6.5 - 40.5)	1.85 ± 1.0 (0.6 - 4.1)

Musculophrenic vein	Distance between vein and skin surface (cm)	1.09 ± 0.27 (0.47 - 1.42)	1.17 ± 0.31 (0.73 - 1.83)
	
	Diameter of vein (cm)	0.73 ± 0.28 (0.28 - 1.32)	0.73 ± 0.29 (0.40 - 1.48)
	
	Maximum blood flow velocity (cm/s)	99.67 ± 44.0 (30.0 - 189.5)	88.91 ± 30.9 (35.6 - 126.8)
	
	Minimum blood flow velocity (cm/s)	6.21 ± 4.9 (0.8 - 18.4)	8.06 ± 7.9 (1.7 - 32.9)
	
	Mean maximum blood flow velocity (cm/s)	65.89 ± 36.2 (15.1 - 136.3)	58.51 ± 27.2 (18.5 - 94.5)
	
	Blood flow volume (l/min)	0.93 ± 0.80 (0.0 - 3.1)	0.67 ± 0.20 (0.2 - 1.0)

## Discussion

Lactating cows had significantly higher blood flow velocities in the milk vein than dry cows. The maximum velocity measured in group 2 (10 kg) was 38 cm/s, which was 2.7 times the velocity in dry cows. Interestingly, there was no further increase in velocity with increasing milk yield in group 3. In contrast, the blood flow volume increased linearly with increasing milk yield from 0.79 l/min in dry cows to 1.80 l/min in group 2 (10 kg; 2.3 fold) and to 3.09 l/min in group 3 (20 kg; 3.9 fold). In another study, a cow with a daily milk production of 40 kg had a blood flow volume of 4.98 l/min in the right milk vein [7], supporting the linear relationship between milk yield and blood flow volume in that vein. Milk yield also affected the blood flow velocity in the musculophrenic vein, but variations were large and the only significant differences were seen in the mean maximum blood flow velocity. This increased 2.7-fold from 30.59 cm/s in the dry cows to 81.90 cm/s in group 2, which was similar to the value in group 3 (85.15 cm/s). The blood flow volume also increased in the musculophrenic vein with increasing milk yield but the differences were not significant. It is surprising that milk yield had an effect on blood flow variables of a vessel not directly associated with the udder; however, lactation has shown to have an effect on the entire circulatory system [8]. Lactating cows had a two- to three-fold higher cardiac output than dry cows, and because 20 to 30% of the cardiac output in a lactating cow is directed toward the udder, the changes are more pronounced in the milk vein than in the musculophrenic vein [8]. The three-fold increase in the blood flow volume of the milk vein in group 3 compared with the dry cows highlights the importance of this vessel for venous drainage of the udder. A blood flow volume of 3.09 l/min translates into almost 9000 l of blood that circulate through the udder via the two milk veins per day compared with 2275 l in dry cows. Three veins are involved in drainage of the udder [5]: the milk vein carries blood to the cranial vena cava; some blood drains toward the caudal vena cava via the external pudendal vein in the inguinal canal; and some blood drains to the caudal vena cava via the ventral perineal vein in a caudodorsal direction through the ischiadic arch and the internal pudendal vein. Which of these three routes drains the most blood has been controversial, and the exact relative contributions of the veins remain to be determined. The external pudendal vein was considered the most important in one study [9], whereas the milk vein was thought to be the major contributor in another [10]. In a third study, the veins were not ranked [11]. Estimates of the amount of blood required for the production of one litre of milk vary and have included 300 to 500 l [9,11], 400 l [5], 500 l [8] and even up to 600 l [12]. Assuming that approximately 500 l of blood are required for 1 l of milk, approximately 10'000 l of blood are required for 20 l of milk. Our results showed that in a cow producing 20 kg of milk, approximately 8900 l of blood flow through both milk veins in one day, which means that the milk vein is the predominant route of venous drainage of the udder, supporting findings by Kjaersgaard [10]. Based on these calculations, approximately 90% of the venous drainage from the udder occurs via the milk veins. This confirms the well-known adage that large milk veins indicate high milk production [5]. This should also serve as a reminder that the integrity of the milk veins must be preserved and complications such as iatrogenic thrombophlebitis must be avoided.

There were no differences between blood flow variables recorded on the left and right sides of the body. Thus, it appears that venous drainage of the udder occurs in a symmetric fashion and that in healthy cattle measurements on one side are representative of both sides. Bilateral measurements are required to objectively assess the degree of blood flow impairment in cows with a unilateral lesion. For instance, the blood flow volume in the thrombosed left milk vein of a cow was reduced to approximately one third of the blood flow volume on the right (0.79 l/min versus 2.54 l/min) [7].

## Conclusions

This study showed that milk production has a profound effect on blood flow variables of the milk vein and to a lesser extent the musculophrenic vein. This must be taken into consideration in future Doppler sonographic studies of these veins and possibly other vessels. Furthermore, measurements on one side are representative of both sides. In cows with unilateral thrombophlebitis of the milk vein, bilateral measurements serve to estimate the degree of impairment on the affected side.

## Competing interests

The authors declare that they have no competing interests.

## Authors' contributions

UB initiated and planned the study and he prepared the manuscript. EF carried out the ultrasonographic examinations under supervision of UB. Both authors have read and approved the manuscript.

## Author details

Department of Farm Animals, University of Zurich.
